# 

**Published:** 1893-01-07

**Authors:** 


					WITH EXTRA NURSING SUPPLEMENT.
Per Annum, 8s. 6d? post free.
Monthly Farts, Sd.j post free, 8d.
Ditto per Annum, 8s., post free.
No. 328. Vol. XTTT.] Saturday,
January 7th, 1893.
[Twopence.

				

